# Severe Infraclavicular Displacement of a Proximal Humerus Fracture with Concomitant Olecranon Fracture in a Pediatric Patient

**DOI:** 10.1155/2022/8673859

**Published:** 2022-02-04

**Authors:** Andrew Shieh, Jessica C. McMichael, Chloe Knudsen-Robbins, Seth L. Brindis, Theodore W. Heyming

**Affiliations:** ^1^Children's Hospital of Orange County, Orange, CA, USA; ^2^University of Pittsburgh School of Medicine, Pittsburgh, PA, USA; ^3^Department of Emergency Medicine, University of California, Irvine, CA, USA

## Abstract

Proximal humerus fractures with severe medial displacement of the humeral head are rare in adults and especially so in children. Concomitant vascular/neurovascular injury is even more uncommon but must be considered as the associated complications can carry significant morbidity. We present a case of a 12-year-old transported to the ED after a mountain bike accident in which she lost control and hit a cement wall, injuring her left upper extremity (LUE). Despite a normal vascular/sensory exam, imaging demonstrated a left comminuted proximal humerus fracture with the humerus displaced medially into the thoracic inlet as well as concern for left subclavian vessel injury. Given the possibility of vascular injury and the severe humeral head displacement, she was taken to surgery with orthopedic and vascular surgical teams. Although surgical exploration did not reveal vascular injury, this case underlines the importance of maintaining a high index of suspicion for neurovascular injury with such fractures as prompt intervention may reduce the likelihood of complications. This case also underscores the need for further research into the management of pediatric proximal humerus fractures in early adolescence.

## 1. Introduction

Proximal humerus fractures are relatively uncommon in children and represent less than 3% of pediatric fractures [1]. In children, proximal humerus fractures typically occur secondary to high-energy direct blows such as those sustained in motor vehicle accidents, sporting injuries, or falls. These fractures have traditionally been treated nonoperatively due to the extensive remodeling potential of the proximal humerus in skeletally immature children [2]. However, the degree of displacement and increasing age are both important considerations when determining the management and potential benefit of surgical intervention. Displacement of the humeral head into the thoracic region is very rare in adults and extremely so in children, and as such, there is debate regarding the necessity of retrieving the humeral head; cases have been successfully treated both surgically and conservatively [3]. Thoracic displacement of the humeral head does raise concern for the possibility of neurovascular injury, and this must be carefully considered. We present a unique case of a skeletally immature child with a proximal humerus fracture with displacement of the humeral head into the thoracic inlet.

## 2. Case Presentation

**Figure 1:**
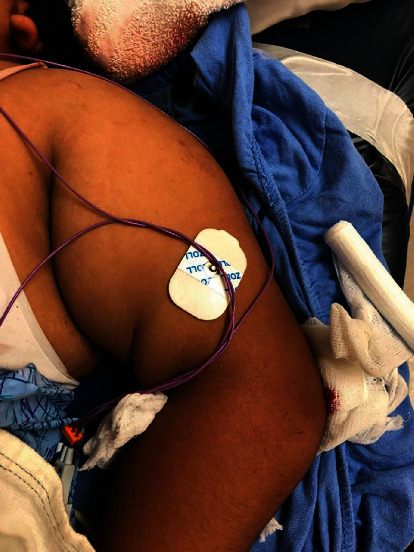
Preoperative photo of the patient's left arm.

A 12-year-old obese female with no significant past medical history was transported via helicopter to the Emergency Department (ED) following an electric mountain bike accident. She stood 1.55 m tall, weighed 108 kg, and had a body mass index of 45 kg/m^2^. The patient reported she was riding down a steep single-track trail at an unknown speed when she lost control and struck a cement wall with the left side of her body. Her left arm bore the brunt of the direct impact as she attempted to brace herself with an outstretched arm. On presentation, she complained only of left upper extremity (LUE) and lower back pain. Her physical exam was significant for limited range of motion (ROM) of the LUE, swelling to the left shoulder, a 0.5 cm abrasion just proximal to the olecranon, soft and depressible compartments, and a normal vascular, motor, and sensory exam (Figure 1).

**Figure 2:**
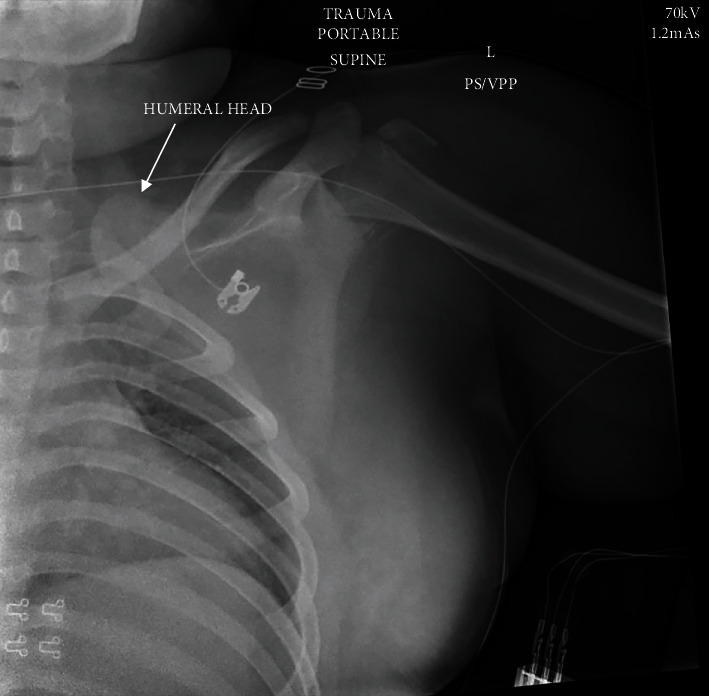
Preoperative anteroposterior X-ray of the left shoulder showing proximal humerus fracture with the humeral head sheared off and displaced medially in the region of the first rib and medial clavicle. Comminuted fragments are noted around the proximal humeral metaphysis.

**Figure 3:**
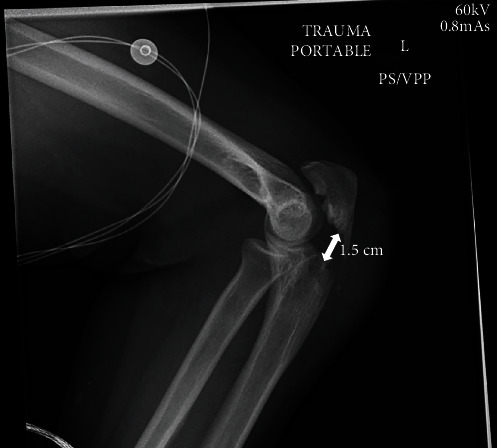
Preoperative lateral X-ray of the left elbow showing fracture of the olecranon process with displacement of 1.5 cm.

**Figure 4:**
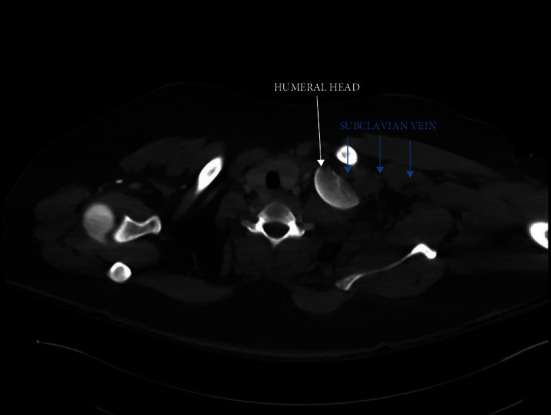
Preoperative CT scan of the chest with contrast confirms 4-part fracture of the proximal humerus with sheared off humeral head displaced posterior to the medial left clavicle above the first rib. The left subclavian vein is attenuated.

Radiographs demonstrated a left comminuted proximal humerus fracture with the humerus sheared off at the anatomic neck and displaced medially into the thoracic inlet, posterior to the medial left clavicle and above the first rib, as well as a displaced, intra-articular left olecranon fracture (Figures 2 and 3). Due to the significant displacement of the humeral head into the thoracic cavity, the arm was immobilized with the elbow by the side for comfort without skeletal traction. A computed tomography (CT) scan of the chest subsequently revealed concern for left subclavian vessel injury (Figure 4). Given the possibility of vascular injury and the severe humeral head displacement, the patient was taken to surgery attended by both orthopedic and vascular surgical teams.

Intraoperatively, the clavicle was exposed via a supraclavicular incision. A chevron osteotomy was made medially and laterally, and the central segment was rotated inferiorly on the pedicle of the pectoralis major. After ligation of a small crossing vascular branch, a small portion of bone was visualized just deep to the vascular branch. Retraction of the soft tissues revealed the humeral head fragment nested in the subclavian tissue. This was removed atraumatically, and the clavicular fragment was replaced and fixed using dynamic compression implants across the osteotomies. The incision was closed with sutures. No major vascular injury was found.

An open reduction of the proximal humerus by the deltopectoral approach was then performed. Within the (significant) fracture hematoma, displaced large fragments of the proximal humerus were visualized. There was significant degloving of the proximal humeral diaphysis down to the deltoid tuberosity. Traction sutures were placed into the rotator cuff at the lesser and greater tuberosities, and the socket was debrided. The humeral head was placed within the wound, and an open reduction of the head to the lesser tuberosity (which contained a small head fragment) was performed. The head was then brought to the shaft using the lesser tuberosity, and a minifragment plate was attached over the lesser tuberosity to secure the head and lesser tuberosity to the diaphysis. The greater tuberosity was then brought around using suture tension and a short plate with locking caps. The plate was secured to the diaphysis, and sutures were placed through the plate holes to neutralize the forces of the rotator cuff.

**Figure 5:**
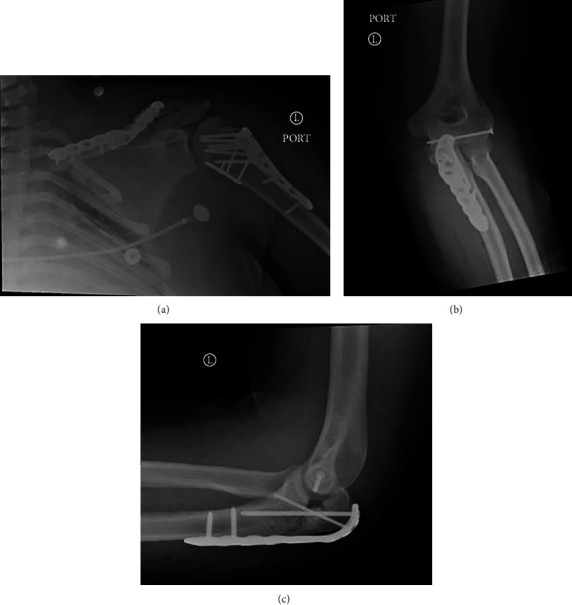
(a) Postoperative anteroposterior X-ray of the left shoulder demonstrating plate and screw fixation of the left clavicle and proximal humerus. (b) Anteroposterior and (c) lateral X-ray of the left elbow demonstrating plate and screw fixation of the proximal humerus and olecranon process.

Repair of the open olecranon fracture was performed using a posterior approach. The wound was debrided, and a standard longitudinal incision over the eminence of the ulna was made, excising macerated skin over the open wound. Comminuted devitalized portions of bone were removed following irrigation and debridement. An unstable, nondisplaced, lateral condyle fracture was found and repaired with a lag screw placed across the lateral epicondyle to the trochlea. An open reduction of the olecranon was then performed, wires were used to temporize the fixation, and a plate was placed deep to the triceps tendon in compression using dynamic compression holes. The patient's wounds were closed, and her arm was immobilized in a splint (for postoperative images, see Figure 5).

**Figure 6:**
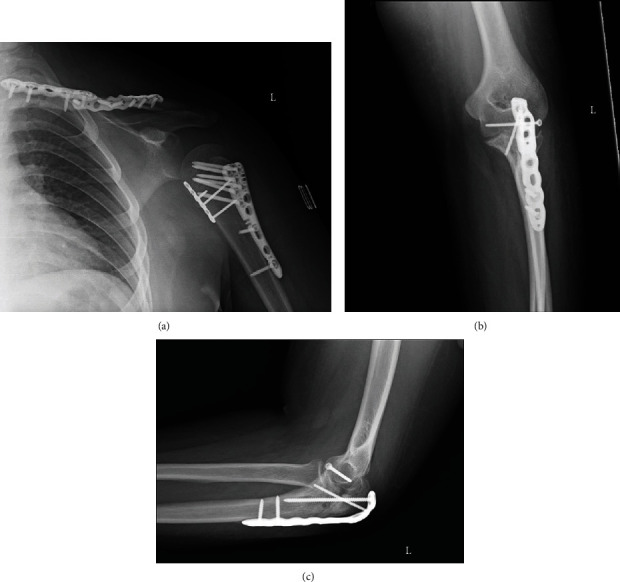
(a) Anteroposterior X-ray of the left shoulder, (b) anteroposterior X-ray of left elbow, and (c) lateral X-ray of the left elbow reveal progressive healing of fractures after six weeks postsurgical intervention.

**Figure 7:**
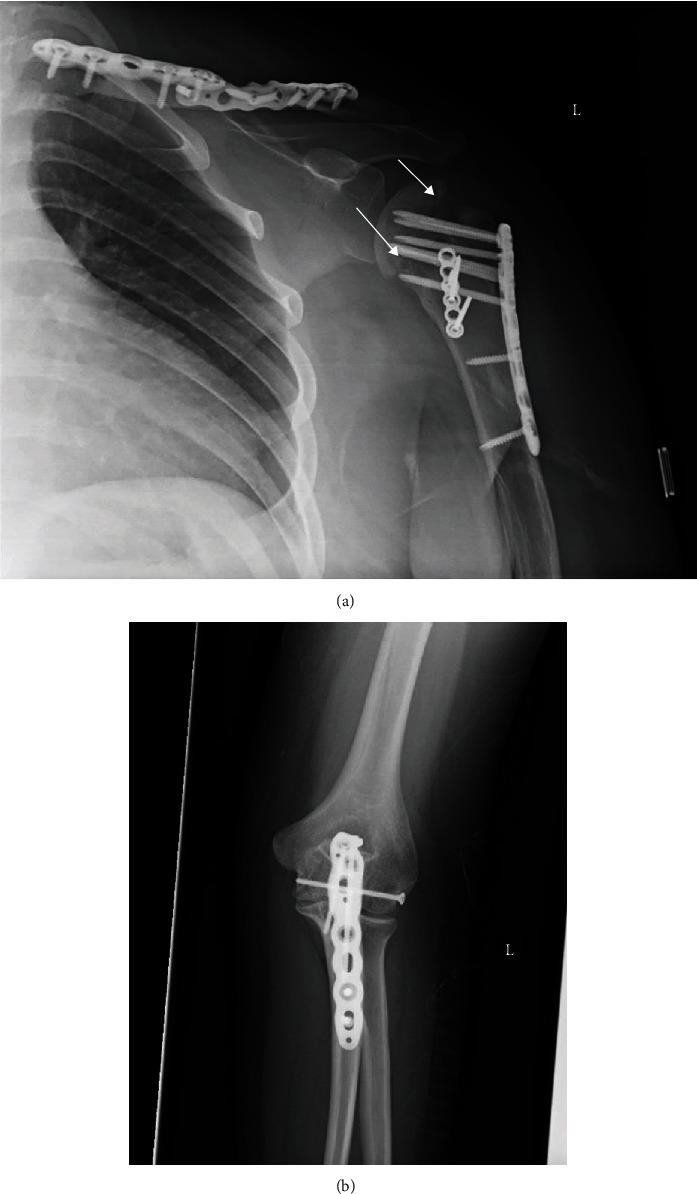
(a) Anteroposterior X-ray of the left shoulder and (b) anteroposterior X-ray of the left olecranon process reveal proper alignment of fractures with subchondral lucency (arrows) seen in the humeral head suggestive of mild avascular necrosis after nine months postsurgical intervention.

She was discharged home four days after surgery, and the splint was removed after six weeks. Radiographs taken at six weeks demonstrated progressive healing changes to the left proximal humerus, clavicle, proximal ulna, and mild humeral head subluxation (Figure 6). The patient began physical therapy, and within nine months of her injury, she had regained full passive ROM with symmetric limb strength. At nine months postsurgical intervention, she continued to have mild pain with active ROM which slowly improved with continued aggressive physical therapy. Her active ROM in the left shoulder and elbow remained limited compared to her uninjuried side. Our patient did not have any numbness or tingling of the shoulder and was able to perform some of her basic daily activities. Radiographs taken at nine months showed evidence of mild avascular necrosis (AVN) of the humeral head, progressive healing, and fracture alignment (Figure 7). Given that the patient continued to have improvement in ROM with rehabilitation one year after the accident, there were no plans to remove the implants electively. However, implant removal could be considered if the patient develops significant pain or impaired function of her shoulder in the future.

## 3. Discussion

Proximal humerus fractures with humeral head displacement into the thoracic space are extremely rare in adults, with less than twenty cases reported in the literature and even more rare in children [3]. Proximal humerus fractures usually occur in high-energy accidents with direct impact to the shoulder, such as in motor vehicle accidents, in children compared to low-energy accidents in the elderly population. Although a direct blow to an externally rotated and extended arm can also cause a shoulder dislocation, proximal humerus fractures are more likely to occur with an adducted limb compared to an abducted arm [1, 3]. We found no cases in the pediatric literature describing a similar humerus fracture and suspect this to be the first documented case of humeral head displacement into the thoracic inlet in a child. Due to the infrequent nature of this injury, definitive management, even in the adult population, has yet to be elucidated [4]. Fracture and dislocation of the humeral head pose significant risk for further thoracic injury due to fragment migration, and surgical retrieval has been shown to be successful in the adult population [5, 6]. There is evidence that CT is the preferred diagnostic imaging modality in these patients as injuries may be missed on plain films and CT offers the opportunity to assess for vascular compromise [5, 6]. In the pediatric case presented here, CT images revealed potential vascular injury, supporting the decision to move forward with surgical repair.

The overall incidence of proximal humerus fractures is approximately 73/100,000, with 75% of these occurring in the elderly and a further subset, 5%, displaying vascular complications [7]. However, management of such fractures is not well studied and there are few reports in the literature of vascular injury in children with proximal humerus fractures [8–10]. In these cases, the physical exam was suggestive of vascular compromise, with two patients presenting with cool, pulseless arms; one with a cuff gradient between the left and right upper extremities; and one with diminished and thready pulses [8–11]. In adults, the physical signs indicating vessel injury can be subtle and can include fractures of the clavicle, scapula, or ribs. Other findings include periclavicular hematomas, neurovascular deficits, and pain or contusions around the shoulder joint [12]. Physical exam has been found to be highly predictive of vascular injury, although the presentation can be delayed, as described by Sandiford et al. with symptoms occurring up to forty-eight hours later [7]. As there is significant morbidity associated with delays in diagnosis and treatment of such injuries, it is essential to carefully evaluate patients at risk for vascular injury [7, 9].

Pediatric proximal humerus fractures account for less than 3% of pediatric fractures with a childhood peak incidence between ten and fourteen years of age [1, 13]. The proximal humeral ossification can be used to estimate skeletal maturity, and it is estimated that the physis closes between ages fourteen to seventeen years in girls and sixteen to eighteen years in boys [1–3, 15, 16]. Historically, humerus fractures without significant angulation or displacement have been treated conservatively in skeletally immature patients due to the extraordinary remodeling potential in skeletally immature patients [2]. However, remodeling potential is age-dependent and while excellent outcomes have been seen in nonoperative treatment of even severely displaced/angulated fractures in patients under the age of eleven years, there is debate as to the treatment of severely displaced/angulated proximal humerus fractures in children because absolute criteria for the degree of displacement as an indication for surgical fixation have not been well described [2, 3, 14]. Surgical risk and complications, including infection, growth plate injury, fractures secondary to hardware fixation, and postoperative limited ROM, must be balanced against the remodeling potential and the goal of return to preinjury upper extremity function. Studies have shown excellent outcomes following surgical intervention in children over twelve with proximal humerus fractures that were closed and displaced without concern for neurovascular injury [17, 18]. The patient's age in this case placed her into a category in which the risks and benefits of surgical reduction/repair remain uncertain, especially when there was concern for vascular injury. Several studies examining outcomes following surgery in patients with severely displaced proximal humerus fractures have demonstrated excellent outcomes, and one study noted that for patients with Neer Classification grade IV fractures, an open surgical approach may be recommended [18–21]. Furthermore, there is a greater occurrence of residual deformity and function limitations in older patients and a more aggressive approach is generally warranted in skeletally mature children [17–21].

One surgical complication of particular concern in patients with proximal humerus fractures is avascular necrosis. Studies in adults, in whom surgical repair is more common, suggest that the number of fracture fragments is associated with an increased risk of AVN, as is poor fracture reduction [22, 23]. The association between timing of surgery and risk for AVN remains equivocal with one study demonstrating an increased risk for AVN following surgeries which occurred more than forty-eight hours postinjury and another demonstrating no correlation between timing of surgical intervention and AVN in patients with surgery occurring before or after seventy-two hours postinjury [22, 23]. Of note, Schnetzke et al. demonstrated that in patients with anatomical neck fractures such as in this patient, surgical repair with a locked plate improved clinical outcomes [23]. Functional outcomes have also been noted to be good in both physeal and metaphyseal closed proximal humerus fractures treated with intramedullary nailing [24]. In our case, the patient underwent surgery within twenty-four hours of the injury and yet, imaging at nine months demonstrated concern for mild AVN. However, our patient has since suffered limited sequela and surgical revision has not been indicated. We recommend all patients with similar shoulder and arm injuries to be closely followed for return of preinjury function and assessed with self-report questionnaires frequently at follow-up appointments.

Our patient's case also highlights the need for increased safety education and regulation regarding use of electric vehicles to prevent pediatric fractures. Injuries in children resulting from electric bikes, all-terrain vehicles, and motor vehicles can result in significant injuries and medical costs.

## 4. Conclusion

Proximal humerus fractures with severe medial displacement of the humeral head are rare in adults and especially so in children. This case highlights the need for more research and establishment of best practice guidelines regarding the management of pediatric proximal humerus fractures, especially with respect to age and displacement parameters as indications for surgical intervention. Although surgical exploration in this case did not reveal vascular injury, this case underlines the importance of maintaining a high index of suspicion for neurovascular injury with such fractures as prompt intervention may reduce the likelihood of complications. Children and parents should know the capabilities of their electric vehicles and follow appropriate safety rules to avoid serious injury.

## Data Availability

The data used to support the findings of this study are included within the References section of this article.
